# Endovascular repair combined with staged drainage for the treatment of infectious aortic aneurysm: a case report

**DOI:** 10.1186/s12872-020-01694-9

**Published:** 2020-09-07

**Authors:** Zhaoxiang Zeng, Zhenjiang Li, Yuxi Zhao, Junjun Liu, Jiaxuan Feng, Zaiping Jing, Rui Feng

**Affiliations:** 1 Department of Vascular Surgery, Changhai Hospital, Navy Medical University, Changhai Road 168#, Yangpu District, Shanghai, China; 2 Department of Vascular Surgery, the First Affiliated Hospital of the Medical School of Zhejiang University, Hangzhou, China; 3 Department of Vascular Surgery, the Affiliated Hospital of Qingdao University, Qingdao, Shandong China

**Keywords:** Infectious abdominal aortic aneurysm, Aortitis, Pseudoaneurysm, EAVR, Case report

## Abstract

**Background:**

Infectious aortic aneurysm, defined as a focal dilation of an infectious arterial wall, is an uncommon life-threatening disease. Compared with open surgery, endovascular repair yields acceptable clinical outcomes. However, residual tissue infection may increase the risk of secondary intervention. Here, we present a successful case of endovascular repair combined with staged drainage for the treatment of infectious aortic aneurysm.

**Case presentation:**

A 58-year-old man presented to hospital with a 3-day history of lower back pain radiating to the back associated with fever. The dynamic imaging characteristics revealed rapid progress of infectious abdominal aortic aneurysm with negative blood culture. The patient underwent endovascular repair and salmonella enteritidis was identified through drain culture.

**Conclusions:**

Endovascular procedure and staged drainage can be feasible and effective option in selected cases.

## Background

Infectious aortic aneurysm (or mycotic aortic aneurysm) is defined as a focal dilation of an infectious arterial wall. It is an uncommon life-threatening disease [1]. It is essential to establish an early diagnosis of infectious aneurysms for timely treatment to improve survival and long-term prognosis. Radiological imaging is critical for detecting initial aortic infection. However, the changes in early images may be minimal, making them easy to be neglected or mixed up [2]. We reported a case with complete sequential typical images of infectious abdominal aortic aneurysm in different stages recorded by series computed tomography (CT) scans, and the patient was successfully treated by endovascular procedure and staged drainage.

## Case presentation

**Fig. 1:**
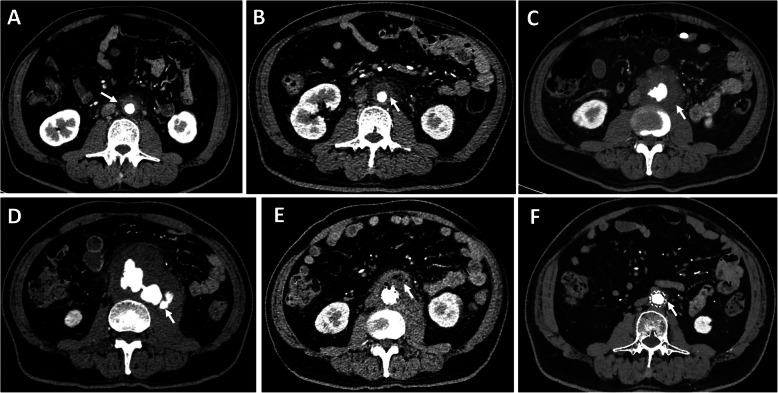
CTA showed the dynamic changes (arrow) of infrarenal abdominal aorta: **a** a periaortic fat stranding, **b** a hypo-attenuating rim, **c** irregular soft-tissue mass, **d** a lobulated pseudoaneurysm, **e** postoperative left psoas muscle abscess and periaortic abscess with gas collection, and **f** normal image. CTA = computed tomography angiography

A 58-year-old man presented to the emergency department with a 3-day history of lower back pain radiating to the back associated with fever (39.7 °C). The physical examination was unremarkable. He has a medical history of diabetes and current cigarette smoking. On admission, enhanced computed tomography angiography (CTA) revealed a moderate atherosclerotic infrarenal abdominal aorta with thickened aortic wall and periaortic fat stranding (Fig. 1a, 2a). A laboratory investigation showed that leukocyte count was normal, while the ration of lymphocytes decreased to 18.2% and eosinophil count was 0. The erythrocyte sedimentation rateand C-reactive peptide were elevated (34 mm/h and 138 mg/L). The serum IgG4 level was within the normal range. The standard bacterial and mycobacterial blood cultures were negative. Nevertheless, infectious abdominal aortic disease was suspected, and intravenous Ciprofloxacin Lactate (0.4 g/d) was administered empirically.

**Fig. 2:**
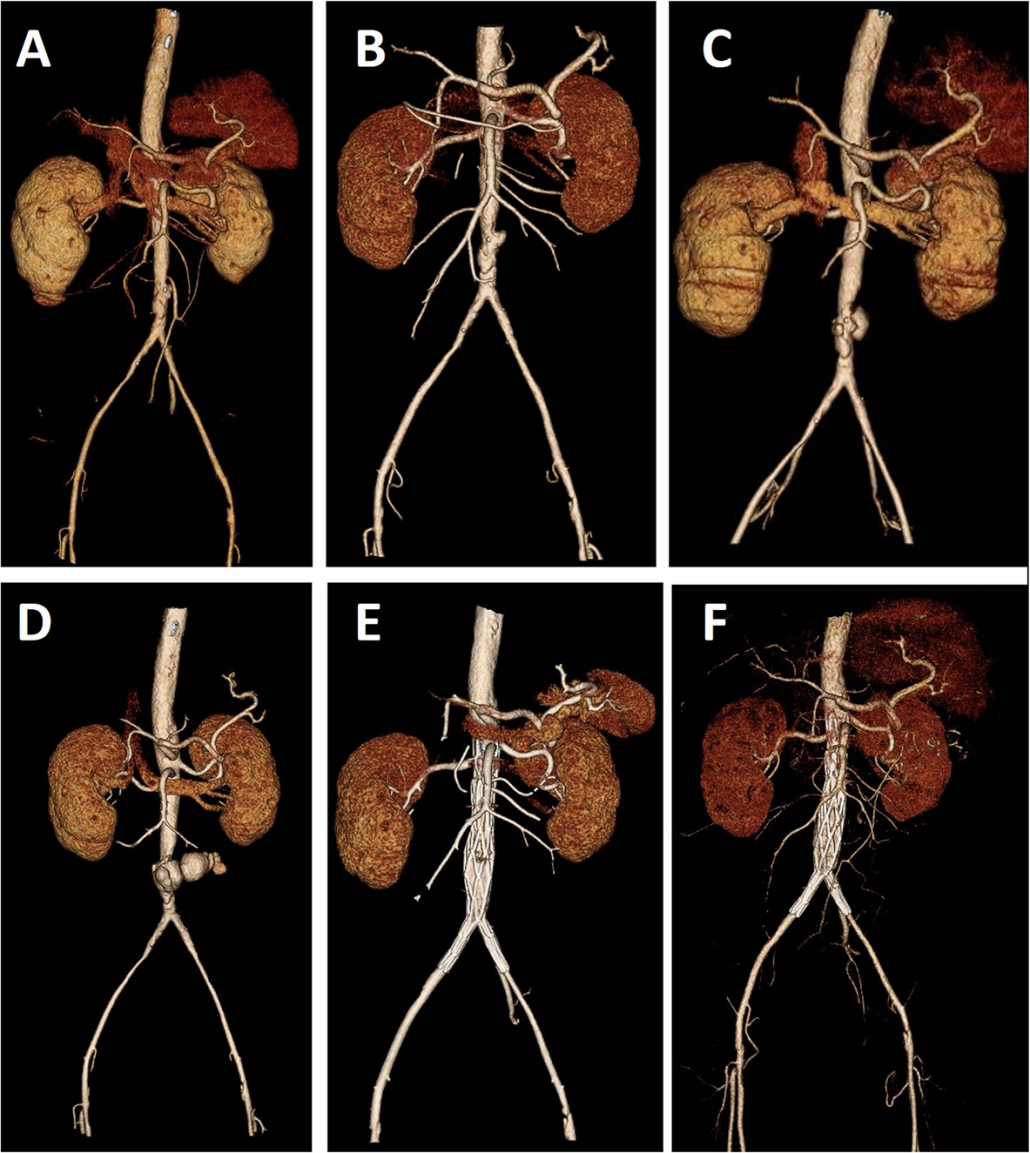
CTA reconstruction of the infrarenal abdominal aorta on day 1 (**a**), 5 (**b**), 21 (**c**), 40 (**d**), 14 days after EVAR (**e**) and 1 year after operation (**f**), showing the dynamic changes of infectious aortic aneurysm. CTA = computed tomography angiography

**Fig. 3:**
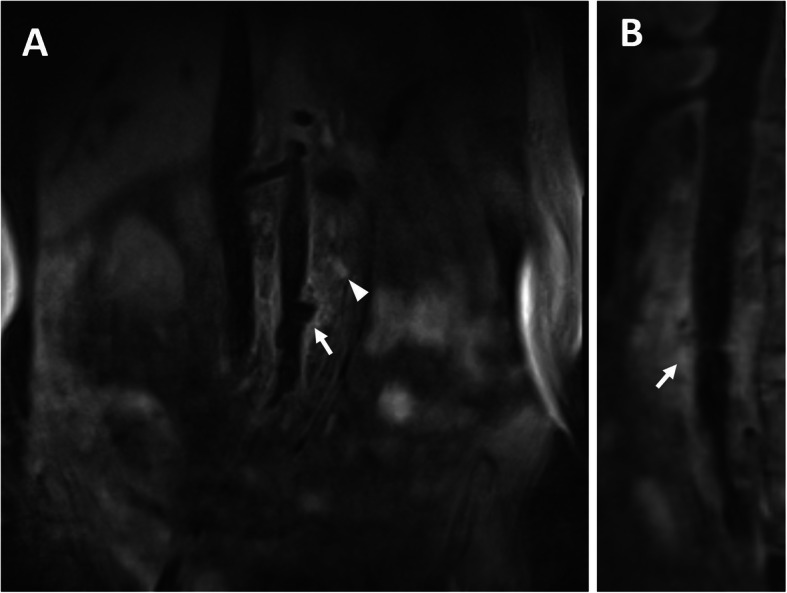
MRA showed high signal intensity in the thickened aortic wall (arrow) and enlarged lymph node (arrowhead) in coronal section (**a**) and sagittal section (**b**). MRA = magnetic resonance angiography

The persistence of back pain and biological inflammation led to of a CTA on day 5. It revealed an enlargement of periaortic infiltration with a hypo-attenuating rim (Fig. 1b, 2b). A magnetic resonance angiography of abdominal aorta showed thickened and enhanced aortic wall, and multiple inflammatory retroperitoneal small lymph nodes consistent with the diagnosis of aortitis (Fig. 3). The abdominal ultrasound revealed uneven thickening of the aortic wall and paraaortic soft-tissue mass with vague margin. The procalcitonin value was 0.446 ng/mL, while the second time standard bacterial and mycobacterial blood cultures were still negative. On day 21, lower back pain got even worse with severe fever (40 °C). CTA showed a saccular aneurysm of 23.6 mm (Fig. 1c, 2c). The ultrasound images of abdominal aorta showed that the aortic wall locally bulged outward along with a thickened discontinuous aortic wall. Biological investigations revealed a high level of leukocyte count, C-reactive protein, erythrocyte sedimentation rate and procalcitonin (1.1 ng/mL). CTA on day 40 showed a lobulated pseudoaneurysm surrounded by enlarged mass with a left psoas muscle abscess (Fig. 1d, 2d). Considering the risk of rupture, we performed an emergency endovascular aortic repair (EVAR) and successfully excluded the aneurysm.

Fourteen days after EVAR, excision and drainage were performed for left psoas muscle abscess and periaortic abscess with gas collection (Fig. 1e, 2e). The Salmonella enteritidis was found in the bacterial culture, which confirmed the diagnosis. Based on bacterial sensitivity tests, intravenous Panipenem/Betamipron was administrated for 2 weeks. The patient recovered with normal imaging (Fig. 1f, 2f) and discharged uneventfully. The patient was regularly followed up annually, and the CTA at 2 years was normal.

## Discussion and conclusions

Infectious aortic aneurysm is uncommon, representing about 0.7% of all aortic aneurysms. But it is a life-threatening condition because these aneurysms tend to progress rapidly and are prone to rupture with high mortality [3]. Diagnosis of infectious aortic aneurysm is also a challenge for nonspecific symptoms and low positive rate of blood culture [4]. CT is considered the first-choice imaging modality as it is available. However, the technetium-99 m/indium-111-labeled leukocyte imaging has been shown to have higher sensitivity in infectious aneurysms. The adjacent soft tissue infections increase the probability of false positivity [5]. In this case, the diagnosis was delayed because the blood culture was persistently negative, but was suspected from the rapid progression on imaging and finally confirmed by identification of Salmonella enteritidis in the paraaortic abscess drainage fluid.

The conventional treatment of infectious aortic aneurysm is usually bimodal with intensive antibiotic therapy as well as surgical repair consisting of resection of the aneurysm, extensive local debridement, and revascularization by insitu reconstruction or extra-anatomic bypass. However, endovascular treatment appears to be a feasible and durable treatment alternative, and its role is growing [6]. The major concern is the risk of recurrent sepsis and infection of the endoprosthesis, as the aneurysm is not resected. Residual tissue infection may increase the risk of secondary intervention. In this case, endovascular repair was used as the first stage procedure and staged drainage was performed to remove infectious tissue. The follow-up image revealed a good aortic remolding.

Standard guidelines concerning early diagnosis, optimal intervention timing, and adequate antibiotic therapy are in need to better deal with infectious aneurysm. Awareness and recognition of imaging findings associated with infectious aneurysms are of paramount importance.

Diagnosis of infectious aortic aneurysm is difficult for nonspecific symptoms and low positive rate of blood culture. Close imaging follow up may be helpful in the diagnosis of infectious aneurysms. Endovascular repair combined with staged drainage can be feasible and effective option in selected cases.

## Data Availability

The datasets used and/or analyzed during the current study are available from the corresponding author on reasonable request.

## Abbreviations

CT: Computed tomography
CTA: Computed tomography angiography
EVAR: Endovascular aortic repair
MRA: Magnetic resonance angiography
